# Safe motherhood in crisis; threats, opportunities, and needs: a qualitative study

**DOI:** 10.1186/s12884-023-06202-3

**Published:** 2024-01-02

**Authors:** Sedigheh Moghassemi, Elham Adib Moghaddam, Sahar Arab

**Affiliations:** Counseling and Reproductive Health Research Center, Department of Midwifery, Faculty of Nursing and Midwifery, Golestan University of Medical Sciences(GOUMS), Gorgan, Iran

**Keywords:** Safe motherhood, COVID-19, Pregnancy

## Abstract

**Background:**

The coronavirus disease (COVID)-19 pandemic has affected many aspects of life, including pregnancy, childbirth, and safe motherhood so that pregnancy and childbirth take place in completely novel and unusual conditions for people. Therefore, we aimed to determine the opportunities, threats, and needs of pregnant women during a crisis.

**Methods:**

The present qualitative study was conducted among women who had a history of pregnancy and childbirth during the COVID-19 pandemic period in 2022. The data were collected by conducting face-to-face, semi-structured and in-depth interviews with 20 purposefully selected participants. Interviews continued until data saturation was attained. Data were analyzed through conventional qualitative content analysis based on the Graneheim and Lundman approach.

**Results:**

The data were categorized under three main themes: 1(“Opportunities for safe motherhood in crisis“(2 Sub‑themes), 2) “Threats to safe motherhood in crisis“(2 Sub‑themes), and 3) “Needs for safe motherhood in crisis” (3 Sub‑themes).

**Conclusions:**

Crisis is not always a threat. By developing an awareness of the opportunities, threats, and needs that safe motherhood faced during the COVID-19 crisis, policy makers can identify the existing gaps affecting the health of mothers and take the necessary measures to improve their conditions, experiences, and health in further crises.

## Introduction

Coronavirus disease 2019 (COVID-19) was first identified in December 2019 in Wuhan, China [1] and declared a public health emergency [2] in January 2020 and a pandemic due to its global spread, as declared by the World Health Organization in March 2020 [3]. This epidemic affected many aspects of life, including pregnancy, childbirth, and safe motherhood [4, 5], which occurred in completely novel and unusual conditions for people [6].

The goal of safe motherhood is to ensure that women have a safe pregnancy and give birth to healthy children [7]. Along the path of safe motherhood, performing lifestyle, fertility, and health behaviors; accessing appropriate and best quality pregnancy care and services; and providing a healthy environment to meet the physical and emotional needs of the mother within an appropriate social and economic status are among the critical factors [8] that can be influenced by the epidemic [5].

For example, nationwide quarantine can lead to delays in accessing appropriate and quality pregnancy care and services, which presents a challenge for safe motherhood programs [9]. Furthermore, women’s rights during childbirth are threatened by pressure on staff and resources, restrictions on the presence of companions and doulas, unnecessary interventions, and separation of mother and baby [10].

Awareness about women’s experiences through pregnancy and after childbirth during the COVID pandemic can facilitate the identification of their health needs and is a decisive step toward improving their conditions and experiences. To this end, the present study was conducted to understand the opportunities, threats, and needs of pregnant women during the COVID-19 pandemic crisis.

## Materials and methods

### Design

The present qualitative study was conducted using the inductive content analysis method followed by receiving the code of ethics (IR.GOUMS.REC.1400.337) from ….University of Medical Sciences. The method of qualitative content analysis includes objective and systematic concepts to explain phenomena, and in the inductive approach, the processes of extracting themes from raw data are explained based on valid inference and interpretation [11].

### Participant

The research participants were women who were pregnant during the COVID-19 pandemic or had a history of pregnancy and childbirth during this period, had no known physical or psychological diseases and were fluent in Persian. The volunteer women were purposefully selected from a wide range of ages, number of children, number of pregnancies, employment status, and ethnicity using databases registered in the health centers of …….

### Data collection

Interviews were conducted in person or by telephone based on the participants’ preferences and condition, with consideration given to their physical and mental health. Throughout the interview, the interviewees’ physical readiness and mental health conditions were taken into consideration by the interviewer; in the case that an interviewee was not in favorable condition, the interview was stopped and postponed to another day. All interviews were conducted and audio recorded with the participants’ permission.

The interview started with the question, “Describe your experiences of pregnancy and childbirth during the COVID-19 pandemic.” Then interview continued based on the semi-structured interview guide. It included “How Covid-19 affected on your life as a pregnant woman?”, “How Covid-19 affected on your prenatal care?”. In order to deepen the interview and obtain richer data, probing questions such as “Explain more using an example?” were used based on the participants’ answers. During the interview, field notes describing the context of the interview and body language or tone of voice indicating the participant’s feelings was recorded along with the time of the interview.

Sampling continued until data saturation was reached, and repeated inferential codes were observed after 23 interviews with 20 participants (of whom 15 had given birth and 5 were pregnant). In three cases, the interview was stopped in the middle due to the participant condition and its continuation was postponed to another time. The duration of each interview varied from 20 to 45 min.

### Data analysis

Data analysis was performed concurrently with data collection using the Graneheim and Lundman approach [12]. To this end, the researchers listened to the recorded interviews several times, ensured full comprehension of the participant’s statements, and transcribed them verbatim.

Each interview was considered a research unit. Following the extraction of the primary codes based on their conceptual and semantic similarities, they were placed under appropriate subthemes, which were later classified under main themes [13].

To ensure the accuracy and reliability of the data, four factors of acceptability, reliability, verifiability, and transferability were employed [13]. To confirm the acceptability of the collected data, the participants were asked to review the transcribed interviews and corroborate their authenticity. To confirm the data reliability, an external researcher was asked to review the procedures of collecting data and extracting the primary codes, subthemes, and themes from the transcribed interviews. In this regard, external researchers as well as three experts in reproductive health and midwifery (who were blind to the research procedure) were provided with texts from a number of interviews as well as the related extracted codes and themes. They were asked to check the accuracy of the data coding process and corroborate its verifiability. To observe the transferability of the findings, we tried to present the participants’ quotes objectively.

## Results

**Fig. 1:**
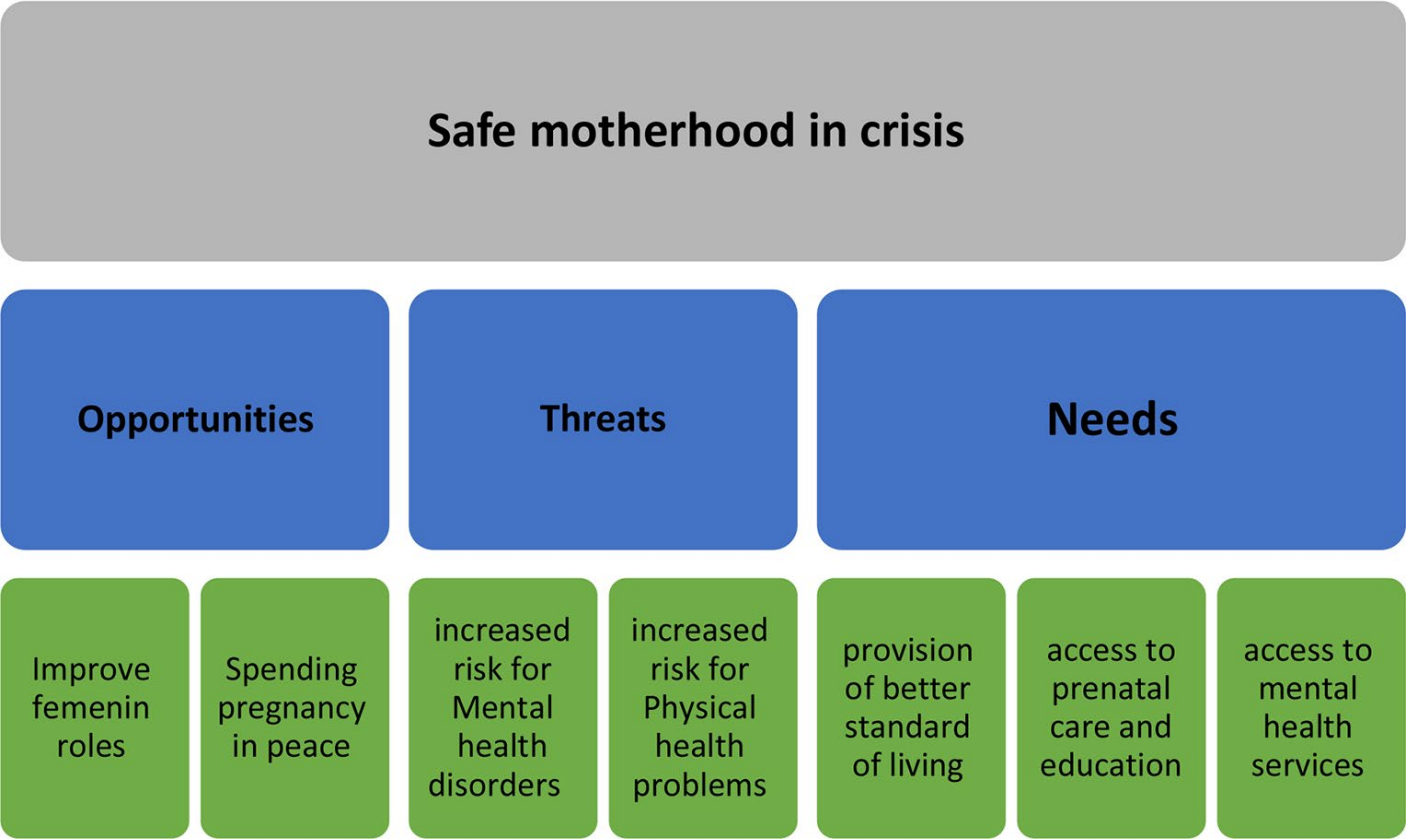
Themes and sub-themes safe motherhood in crisis

The participants’ demographic characteristics are presented in Table 1. After merging repeated codes, the 96 inferential codes were condensed into 22 inferential codes, which were grouped into 7 subthemes and 3 main themes (Table 2) (Fig. 1).

**Table 1:** Demographic characteristics of participants

Participants (No)	Age (years)	Gravidity	Ethnicity	Pregnant/History of pregnancy in COVID	Gestational age at the time of interview	Education level	employment statues
1	31	1	Fars	History of pregnancy	-	university degree	Working
2	31	1	Turkmen	History of pregnancy	-	university degree	Working
3	20	1	Fars	Pregnant	24 Weeks	Secondary school /diploma	Working
4	28	2	Fars	History of pregnancy	-	university degree	Household
5	31	1	Fars	Pregnant	36 Weeks	university degree	Household
6	25	1	Other	Pregnant	34 Weeks	Secondary school/ diploma	Working
7	30	1	Fars	History of pregnancy	-	university degree	Working
8	29	2	Turkmen	History of pregnancy	-	Secondary school/ diploma	Household
9	39	3	Turkmen	History of pregnancy	-	Secondary school/ diploma	Household
10	32	3	Other	History of pregnancy	-	Secondary school/ diploma	Household
11	33	1	Fars	History of pregnancy	-	Secondary school/ diploma	Working
12	36	3	Turkmen	History of pregnancy	-	Secondary school/diploma	Household
13	35	3	Fars	History of pregnancy	-	university degree	Working
14	32	2	Fars	History of pregnancy	-	Secondary school/ diploma	Household
15	34	2	Fars	History of pregnancy	-	university degree	Working
16	29	2	Turkmen	History of pregnancy	-	university degree	Household
17	28	1	Fars	History of pregnancy	-	Secondary school diploma	Working
18	19	G1	Fars	Pregnant	36 Weeks	Student	Student
19	31	G2	Fars	History of pregnancy	-	university degree	Household
20	25	G1	Fars	Pregnant	32 Weeks	Secondary school/ diploma	Working

**Table 2:** The primary codes, sub-themes and themes

Condensed codes	Subthemes f (%)	Main themes f (%)
° More opportunity for self-care ° More opportunity for motherhood ° More opportunities for wifery	Providing more opportunities to improve feminine roles in personal and interpersonal relationships 15 (15.46)	**Safe motherhood opportunities in crisis** **24 (24.74)**
° Staying away from others’ negative impositions ° Staying away from unnecessary crowds ° Staying away from others’ words and judgments	Spending pregnancy in more peace 9 (9.27)	
° Worrying about infection of oneself, the fetus, the newborn baby, and the people around you with the virus ° Feeling emotional paradox about pregnancy and maintaining the fetus ° Feeling negative mood swings ° Reduced opportunity of gaining some maternal experiences ° Experiencing psychological pressure caused by social control for pregnancy ° Experiencing neglect, social isolation, and psychological pressures caused by the pandemic ° Feeling concerns by working mothers during COVID	Increased risk for Mental health disorders in pregnant women in crisis 40 (41.23)	**Safe motherhood threats in crisis 50 (51.54)**
° Disruptions in maternal and child health care systems ° Adverse consequences associated with compliance with health protocols ° The challenges of working mothers in COVID	Increased risk for Physical health problems in crisis 10 (10.30)	
° Developing public health and life skills education to support pregnant mothers in crisis ° Availability of different types of economic support for pregnant women during the crisis ° Availability of different types of occupational	Improving the living standards of pregnant mothers in crisis 9 (9.27)	**Safe motherhood needs in crisis 23 (23.71)**
° Facilitating reception of prenatal and postnatal care using various strategies ° Providing a safe environment to receive maternity and child care during crisis	Improving the pregnant women access to pregnancy and childbirth related education and care during crisis 10 (10.30)	
° Providing psychological services during and after pregnancy in different ways (in-person, Virtual, etc.)	Improving the access of pregnant women to mental health services 4 (4.12)	

f: Frequency of repetition

### Safe motherhood opportunities in crisis

The pandemic provided pregnant women with opportunities and positive experiences.

#### Providing more opportunities to improve feminine roles in personal and interpersonal relationships

In this regard, they mentioned more opportunities for self-care, motherhood, and wifehood. One participant said:

I had more time for myself at home, I could read more, meditate, and even spend more time with my baby; I had a great time. (P 7)

Another participant mentioned that how spending time with his husband make her relationship better. She said “My husband and I had the opportunity to spend more time together that improved our relationship; we watched movies together and talked more. This makes me feel good when I think about those days. (P 4).

#### Spending pregnancy in more peace

Pandemic conditions caused mothers to stay away from unnecessary crowds, avoid other people’s negative impositions, words, and judgments, and spend their pregnancies more peacefully.

One participant said:

COVID was a positive experience for me because I was away from the crowd and unnecessary parties. I spent my pregnancy in silence and peace away from the hustle and bustle of this era. (P 15).

A women with previous history of abortions said:

Since I had several miscarriages before, I was worried that everyone would know that I was pregnant and would judge me in the case of another miscarriage. However, I did not have to worry about this due to the COVID pandemic. (P 10).

### Safe motherhood threats in crisis

The pandemic outbreak created threats to the physical and mental health of pregnant women.

#### Increased risk for mental health disorders in pregnant women in crisis

The participants reported negative mood changes; worries about themselves, the fetus, the newborn baby, and others’ infection with COVID; emotional paradoxes about pregnancy and maintenance of the fetus; reduced opportunities to gain some maternal experiences; psychological pressure caused by social control for pregnancy; feelings of being ignored and socially isolated; and the mental pressures caused by such feelings as threats to mental health and disruption in maternal and child health care systems.

One of primiparous women said:

Isolation may be considered the best solution in dealing with COVID, but being isolated during pregnancy was not good for me at least. I was pregnant, and social isolation was the worst possible thing that could occur. Because I needed mental and psychological varieties; I needed to go out. I needed family support and social interaction since I used to deal with a lot of people in my job. Given that in every family, there are people with underlying diseases, I was worried that I may not be welcomed in my mother or mother-in-law’s house. Therefore, I could not get along with the mental and emotional burden caused by my pregnancy since I was not treated like a pregnant woman. Because it was my first experience in pregnancy, I liked it to be spent in a better situation. (P 2).

When people in the community saw that I was pregnant, they said “take care of yourself”, hearing this did not create a good feeling because even if I wanted to be comfortable, others would imply that I had to be stressed. They said “what have you done”, and it even made me feel ashamed about my pregnancy. (P 2).

#### Increased risk for Physical health problems in crisis

Disruptions in the healthcare system, the adverse consequences related to compliance with health protocols and the challenges of working mothers were raised as threats to their physical health.

It was very difficult to wear a mask during pregnancy because I felt short of breath and using a mask made the situation more painful, especially when I was wearing two masks at work, sometimes I felt like I could not catch my breath. (P 17).

In my opinion, it was more important not to get infected with the virus than to receive prenatal care. That is why I preferred to stay at home and avoid having high-risk contacts than to go to check my blood pressure and fundal height. Although I referred to the health center for tetanus vaccination, they did not record my household information in the electronic health system. However, after giving birth, they blamed me for not having an electronic profile and so do not perform the child’s thyroid test. (P 16).

### Safe motherhood needs in crisis

#### Improving the living standards of pregnant mothers in crisis

Participants indicated that mothers need measures to improve their standard of living when pregnancy and childbirth are spent in crisis. For instance one participant mentioned

Considering the economic problems caused for the public during the pandemic (due to the quarantine), they could have considered some economic support programs such as providing main groups food packages or allowances for essential procedures, such as ultrasound for the pregnant women and new mothers. (P 14).

#### Improving the pregnant women access to pregnancy and childbirth related education and care during crisis

The participants raised the need to facilitate receiving child and maternity services and provide a safe environment for mother and child care during the crisis. One participant said

My greatest question, which was not responded to, was ‘Why they did not allocate a specific ultrasound center or laboratory for pregnant women?’ We had to refer to the centers where patients with coronavirus disease were referred for diagnosis tests or images. (P 19).

#### Improving the access of pregnant women to mental health services

The participants pointed out the need to improve mental health during the crisis and ways for facilitating access of pregnant women to mental health services. A multigravida woman said

Considering that pregnant mothers are under higher levels of pressure than other people in terms of mental and emotional health during the pandemic, in my opinion, there is a real need to take the required measures to provide this peace. To this end, educational pamphlets or short movies should be developed to calm down pregnant women so that they are not affected by negative thoughts. (P 13).

A primigravida participant mentioned:

I think that the health centers could have contacted us to give some information, reassurance, and training to reduce our fears and worries. This could have been very helpful because the worries and concerns of a pregnant woman are certainly different from those of ordinary individuals in a society. I believe the health authorities could have set up a special telephone line, a contact number, or a text message number to be used by pregnant mothers when they felt worried or anxious about the pandemic. If they truly did so, I think a better pregnancy experience could have been created during the COVID period. (P 1).

## Discussion

The mothers investigated in the present study noted the quarantine and special conditions of the COVID-19 epidemic as an opportunity to improve their womanliness as well as personal and interpersonal relationships under self-care, motherhood, and wifehood. They also experienced more peace by staying away from crowds, negative impositions, and other people’s judgments.

A large number of studies conducted on the experiences of pregnant women and new mothers during the COVID-19 pandemic have focused on the negative effects of quarantine [14–17], while the current study also revealed the positives, which are seen as an opportunity for safe motherhood in the crisis.

Some studies carried out among nonpregnant women during the COVID-19 pandemic have clarified opportunities similar to those of the present study.

Some female faculty members of Turkish universities described the quarantine period as an opportunity to increase the time spent with family members, get to know them better, and bond with them. The participants mentioned that during the quarantine, they could focus on observing the growth of their children. Some of them also mentioned that the days of quarantine had a positive effect on their family relationships [18].

Based on the lived experiences of married women in Tehran, Pourmousavi et al. identified a pattern of opportunities and threats following quarantine. Quarantine provided more time for having conversations between couples, focusing on individual progress, performing joint activities with family members, completing backlogged tasks, having more opportunities for self-care, and reducing tension within the family [19].

Mazumder et al. determined the positive effects of pandemics on the personal and interpersonal relationships of Indian youth. As the participants noted, the quarantine condition provided them with the opportunity to discover other aspects of their personality by doing different hobbies and spending more time with their family members.

Findings shed some light on different aspects of coronavirus disease as a multidimensional phenomenon so that all members of the community, including pregnant women and new mothers, can notify them of the opportunities at the heart of epidemic threats and employ them to meet the related challenges appropriately [20]. Given that crises may occur at any time and remain for any period, identifying the opportunities and living with the necessary precautions, innovations, and improvisations is the only solution [21].

Mothers who participated in the present study also identified threats to their physical and mental health during the pandemic. They experienced mood changes, worries about getting infected, social isolation, psychological pressure caused by social control, and disruption of the health care system as threats.

In line with the present study, Barbosa-Leiker et al. (2021) in the United States reported disruption in receiving family and social support as well as important pregnancy and childbirth care [16]. In the same vein, Sahin and Kabakci (2021) in Turkey observed disruption in routine pregnancy care, disruption in social life, and negative effects on mental health as a threat for pregnant women during the pandemic [15].

Pregnant women in Brazil were concerned about the possible consequences of the virus for the baby, not recovering completely, isolation due to the disease, and the stigma associated with the disease in their community [22]. Thus, the COVID-19 epidemic significantly increased the risk of anxiety in women during the perinatal period, maintaining that the mental health of this susceptible population requires supportive measures in this regard [23].

In Nepal, factors associated with safe motherhood were directly or indirectly affected by the epidemic. Disruption in maternal health care procedures and the low priority of screening for maternal mental health challenges were identified as threats to safe motherhood. Therefore, factors affecting safe motherhood should be considered by applying strategic plans integrating physical and mental health based on evidence in the conditions of the epidemic so that mothers experience a safe and pleasant birth [21, 24].

In the present study, mothers raised unanswered needs during their pregnancy in epidemic conditions, including access to physical and mental health services and improvement of their living standards.

The participants pointed out that the epidemic had affected their access to healthcare services, while taking some measures could have been effective in improving the situation, such as considering some special phone lines for pregnant mothers to call or send messages to receive virtual and remote medical and consulting services. In the case of receiving paraclinical services that require the patients’ physical presence, the participants needed special centers for pregnant women.

In this regard, the results of a study by Goudarzi et al. in Iran illustrated that more than half of pregnant women neither contacted health centers (by telephone) nor received training and screenings related to physical and mental health. In addition, less than 10% of women had received the usual pregnancy care services in full. “Accessibility” received the lowest score among the five subscales of the quality of prenatal services. The existence of restrictions during the epidemic, fear of infection, and limited human and financial resources were suggested as possible reasons for the low quality of prenatal care [25].

Brislane et al. also reported impaired access to health care services among pregnant and postpartum women; 62% of mothers mentioned that COVID-19 had affected their access to health care. According to these scholars, continuity of receiving care in epidemic conditions can be followed-up through virtual and/or telephone appointments [26].

Telemedicine is a useful resource for pregnant women during a pandemic because it reduces the waiting time and the risk of infection by significantly limiting face-to-face visits to the clinic [16]. The development of online prenatal care programs is likely to have economic benefits for the health care system and women in terms of cost, time, and manpower, which can improve overall maternal and reproductive health services and family life [27]. In this regard, pregnant women with COVID-19 in Brazil also expressed the importance of the support of the health team, especially the use of telemedicine to monitor their health conditions during the illness process [22].

When the focus is on positive cases of COVID, policies should be developed to diagnose the early symptoms of mental illnesses that are increasing in non-COVID women during the epidemic [21]. To better support women’s mental health in the perinatal period, health care providers should refer patients to counseling centers and virtual support groups and continuously screen pregnant women for depression and anxiety [16].

The provision of financial assistance for medical care and life necessities was among the other requirements raised by participants of the present study. According to Wu et al., daily needs are scantly satisfied, and medical costs are much higher than usual throughout epidemics. Many family heads may lose their jobs during pandemics, and a heavy economic burden is consequently imposed on pregnant women in various ways [27].

Ahlers-Schmidt et al. reported significant changes in the occupational and financial status of pregnant and postpartum women during the pandemic [28]. Moreover, 43% of the women in Thayer and Gildner’s study reported experiencing financial stress due to the COVID-19 pandemic [29].

Financial burden can double the likelihood of depression during pregnancy, which can affect the outcome of childbirth and the long-term health of children [29]. Barbosa et al. also concluded that during the epidemic, families were faced with financial problems in the provision of basic food items and necessities of life. To meet this problem, assistance should be provided for the provision of powdered milk, diapers, and other supplies needed by mothers and children [16].

## Strengths and limitations

Among the strengths of the current study was the examination of the experience of pregnancy and childbirth during the period of COVID from the point of view of the women themselves. The present study, in addition to identifying the threats and needs of mothers, has also achieved positive points that can be an opportunity for safe motherhood in critical situations.

Qualitative studies have little generalizability by nature, although an effort was made to increase the generalizability of the results with the participation of women with different characteristics. Conducting qualitative studies with the participation of policymakers in the field of maternal and child health can help to identify other aspects of this issue.

## Conclusion

The results of this study emphasize that crises like COVID-19 can be a multidimensional phenomenon with different effects on pregnant women’s quality of life. Pregnant women confront different challenges, threats, and needs along with perception of some positive experiences. The development of a comprehensive awareness of these dimensions can help policy makers focus on opportunities while eliminating threats to meet pregnant women’s needs. Implementation of the above-mentioned policies related to pregnancy during epidemic conditions can facilitate realizing safe motherhood in crisis.

## Data Availability

The datasets used and/or analysed during the current study are available from the corresponding author on reasonable request.
